# Radiotherapy for subependymal giant cell astrocytoma: time to challenge a historical ban? A case report and review of the literature

**DOI:** 10.1186/s13256-024-04649-2

**Published:** 2024-07-20

**Authors:** Randa Kamel, Dirk Van den Berge

**Affiliations:** Radiotherapy Department, UZ Brussel, Laarbeeklaan 101, 1090 Brussels, Belgium

**Keywords:** Subependymal giant cell astrocytoma, Tuberous sclerosis complex, Fractionated stereotactic radiotherapy

## Abstract

**Background:**

Subependymal giant cell astrocytoma is a benign brain tumor that occurs in patients with tuberous sclerosis complex. Surgical removal is the traditional treatment, and expert opinion is strongly against the use of radiotherapy. Recently, success has been reported with the mTor inhibitor everolimus in reducing tumor volume, but regrowth has been observed after dose reduction or cessation.

**Case report:**

We present the case of a 40-year-old Asian female patient treated successfully for growing bilateral subependymal giant cell astrocytoma with fractionated stereotactic radiotherapy before everolimus became available. After a follow-up of 8 years, everolimus was administered for renal angiomyolipoma and the patient was followed up until 13 years after radiotherapy. Successive magnetic resonance imaging demonstrated an 80% volume reduction after radiotherapy that increased to 90% with everolimus. A review of the literature was done leveraging Medline via PubMed, and we assembled a database of 1298 article references and 780 full-text articles in search of evidence for contraindicating radiotherapy in subependymal giant cell astrocytoma. Varying results of single-fraction radiosurgery were described in a total of 13 cases. Only in two published cases was the radiation dose of fractionated radiotherapy mentioned. One single publication mentions an induced secondary brain tumor 8 years after whole-brain radiotherapy.

**Conclusion:**

There is no evidence of contraindication and exclusion of fractionated radiotherapy in treating subependymal giant cell astrocytoma. Our experience demonstrates that subependymal giant cell astrocytoma, as other benign intracranial tumors, responds slowly but progressively to radiotherapy and suggests that fractionated stereotactic radiotherapy holds promise to consolidate responses obtained with mTor inhibitors avoiding regrowth after cessation.

## Introduction

Subependymal giant cell astrocytoma (SEGA) is a non-invasive World Health Organization (WHO) grade 1 glioma that occurs in 20% of patients with tuberous sclerosis complex (TSC), usually in the first two decades of life [1]. They are located mainly in the periventricular area and are bilateral in 20% of the cases [1]. SEGA most frequently occurs in patients with accompanying features of TSC, which is an autosomal dominant neurocutaneous disorder caused by mutation in the TSC-1 or TSC-2 genes that involves brain, skin, eyes, lung, liver, and kidneys. Only 38 cases of SEGA have been described in patients without other clinical features of TSC, but in 7 out of 9 cases in which molecular analysis was performed, a mutation of TSC-1 or TSC-2 was still detected in tumor tissue [2]. Brain involvement of TSC consists of delayed neurocognitive development and growth of benign tumors that are classified into intraparenchymal hamartomas and subependymal nodules. The latter can demonstrate accelerated growth and are then called SEGA, although they remain histologically identical to subependymal nodules [3]. SEGA have been shown to be responsible for 25% of the excess mortality in patients with TSC [4] by causing hydrocephalus and sudden death.

The standard treatment of symptomatic SEGA is complete surgical removal. The only alternative treatment option as of today, pharmacological treatment with everolimus, has been approved by both the European Medicines Agency and Food and Drug Administration only when curative resection is not possible [5].

Recently, research has shifted to pharmacological treatment, as mTOR inhibitors such as everolimus were shown to induce significant responses, and a multicentric randomized, placebo-controlled phase 3 trial (EXIST-1) demonstrated that at least 35% of patients had at least 50% reduction in SEGA volume after 6–9 months of treatment with everolimus [6, 7].

While fractionated stereotactic radiotherapy (FSRT) is a standard treatment modality in the treatment of other low-grade gliomas [UpToDate, Post TW (Ed), UpToDate, Waltham, MA; last accessed on 30 December 2022], no experience with FSRT in the treatment for SEGA has ever been reported. There appears to be broad consensus that no form of irradiation should be used for this indication, and if at all mentioned as a treatment, it has been described as “ineffective” [8], “proved inefficient” [9], and it is claimed that “logically, some kind of radioresistance should be observed” [10]. In the recommendations report of the 2012 International Tuberous Sclerosis Complex Consensus Conference, it is stated (without a supporting reference) that there is a lack of responsiveness to radiotherapy [11]. In the same report, as in many others, concern is expressed about potentially increased risk in TSC of developing secondary malignancies due to radiotherapy and chemotherapy.

We report a case of bilateral SEGA that the referring neurosurgical team decided not to operate on and that was irradiated before mTor inhibitors became available. After a follow-up of nearly 8 years, we observed a dramatic volume reduction of both bilateral tumors, and when everolimus was started for angiomyolipomas of the kidneys, the SEGA volume further decreased.

Motivated by this favorable outcome, we decided to accurately document the volumetric response of the tumor to radiotherapy and later to everolimus, to conduct a systematic review of the literature concerning irradiation of SEGA, and to review the rationale behind the consensus against the use of radiotherapy in the treatment of SEGA.

## Case report

We present a 40-year-old female patient of Asian ethnicity, diagnosed with TSC since childhood on the basis of the typical skin lesions combined with delayed psychomotor and intellectual development. When she was 10 years old, bilateral periventricular hamartomas had been observed on brain imaging; computed tomography (CT) in Fig. 2 gives a detailed overview of both tumor sizes at presentation and throughout the follow-up years. The clinical diagnosis of TSC was subsequently confirmed by the demonstration of a mutation in the TSC2 gene.

The patient was referred to the neurosurgery department at the age of 22 years for worsening headaches and behavioral changes presenting in aggression. At that time, everolimus was not yet in use for treatment of SEGA. A wait-and-scan approach was adopted for about 3 years, and the volume of both SEGA doubled during that time. When she was 25 years old, she suffered from signs of increased intracranial pressure due to obstructive hydrocephalus, and a ventriculoperitoneal drain was placed and symptoms improved rapidly. Due to the presence of bilateral tumors, the absence of sufficient ventricular dilation to facilitate endoscopic resection, and the compromised neurocognitive status, the referring neurosurgeons were reluctant to perform bilateral transfrontal surgery and asked for radiotherapeutic advice.

At that time, very limited data were available concerning radiotherapy for this indication, but it was felt that it was reasonable and safe to prescribe a fractionated stereotactic radiotherapy at a dose of 60 Gy (30 fractions of 2 Gy) to the gross tumor volumes with a 2 mm planning target volume (PTV) margin.

This treatment was well tolerated, did not require prophylactic corticotherapy, and did not cause acute side effects.

**Fig. 1:**
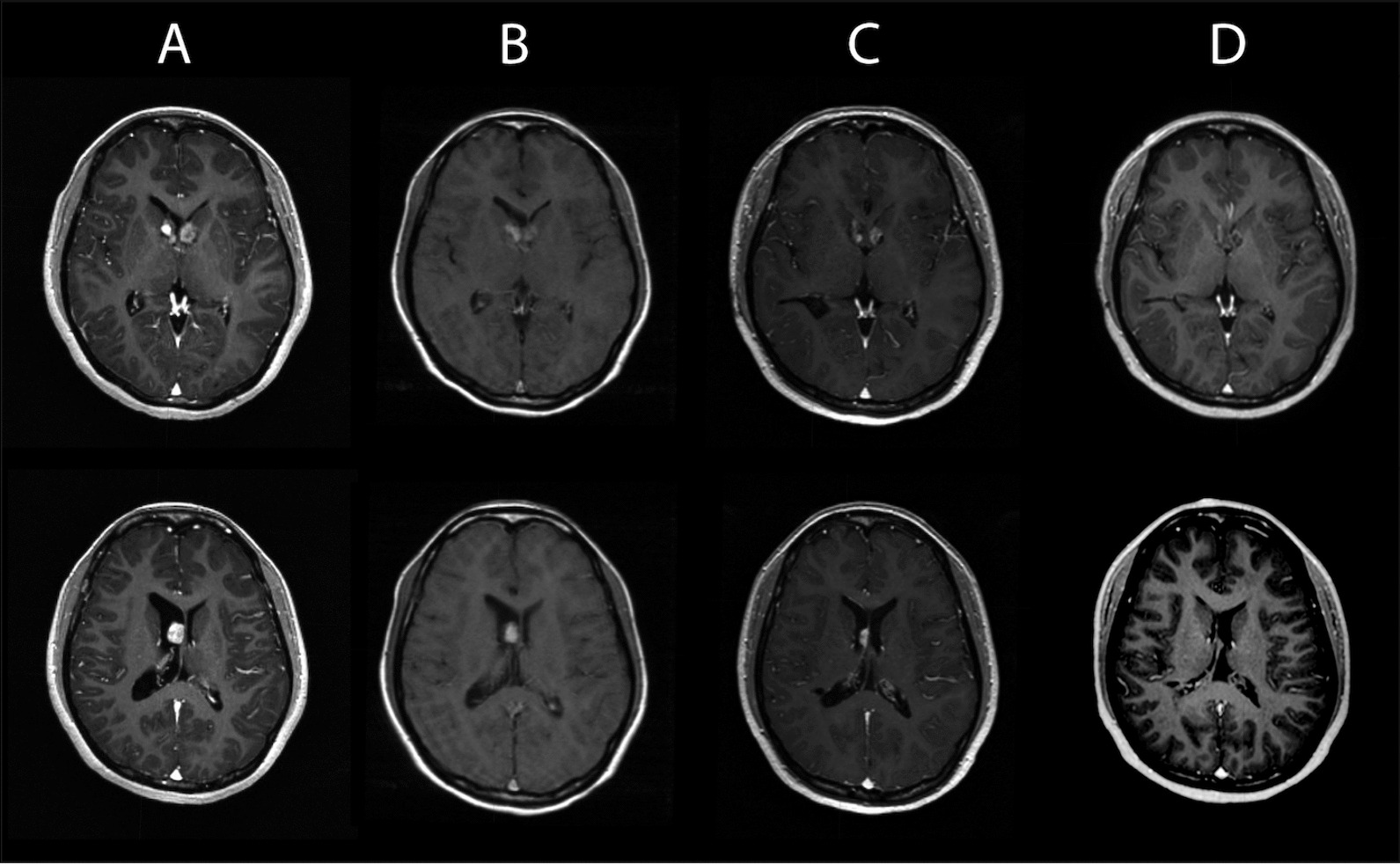
Gadolinium-enhanced T1 images of the left-sided SEGA (upper row) and the right-sided SEGA (lower row). Columns correspond to the timepoints indicated in Fig. 2. **A** Start of radiotherapy; **B** after 4.3 years; **C** after 7.6 years, start of everolimus; **D** after 12.5 years

We followed the tumor response through volumetric assessment through slice-by-slice delineation of gross target volumes (GTVs) on 1 or 2 mm T1 gadolinium-enhanced magnetic resonance imaging (MRI) in the 17 pre- and post-radiotherapy MRIs done over more than 16 years (Fig. 1). A slow tumor regression was observed in the first year, but in the second year after radiotherapy, the SEGA regression accelerated and continued, until nearly 8 years after radiotherapy, their volume had decreased by 72% and 82%, respectively.

**Fig. 2:**
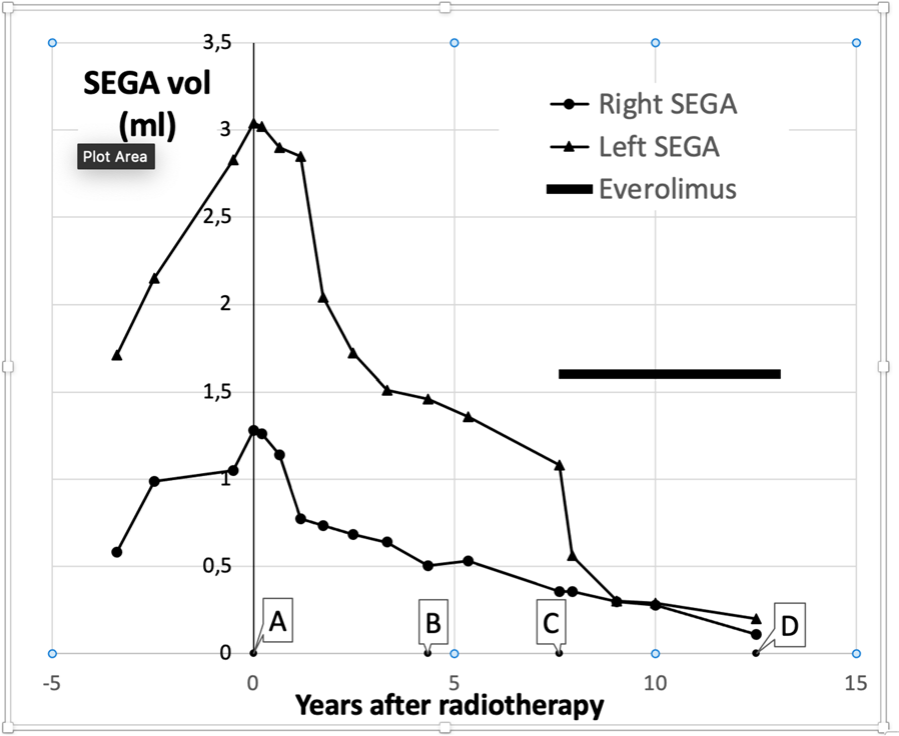
Volume of both SEGAs before and after radiotherapy. Illustrative MRI at the indicated timepoints are provided in Fig. 1. Point A: start of radiotherapy, point C: start of everolimus, point D: current volume

At that point in time, everolimus (2.5 mg/d) was started to treat growing bilateral renal angiomyolipomas. Remarkably, the residue of the larger SEGA responded dramatically to everolimus (Fig. 2), again within 1 year by 50% reduction of its remaining volume, while the speed of volume reduction of the smaller tumor did not seem to be influenced by the drug (Fig. 1). After a follow-up of 13 years after radiotherapy and 5 years of everolimus, both tumors had a residual volume of less than 10%. We never observed signs of increased tumoral or peritumoral inflammation or edema, nor did the patient at any time have seizures.

## Review of the literature

To find publications relevant to the efficacy and toxicity of irradiation of SEGA, we used Endnote 20 to search Medline via Pubmed using the search terms: “treatment of SEGA”, “radiotherapy in SEGA”, “SRS in SEGA”, “treatment of tuberous sclerosis complex”, and “radiotherapy treatment in low grade astrocytomas”. All freely accessible and a selection of charged-access full-text articles were downloaded, and contents were indexed. In a second step, the article references were examined and queried for the terms “radiotherapy”, “induced tumors”, and “malignant tumors”. Secondary references were added to the database and reprocessed the same way as the originally found references. Reviews about secondary radiation-induced tumors in general were added to the database as well. Specific attention was given to potential traces of reports containing data about irradiated SEGA and about high-grade brain tumors in patients with TSC. The database was locked on 12 January 2022, containing 1298 article references and 780 full-text articles.

Finally, all database fields, as well as the indexed content of all full-text articles, were screened to find reports on irradiated SEGA and on malignant brain tumors in patients with TSC as well as radiation-induced tumors in patients with TSC.

### Tumor control with radiosurgery and radiotherapy

There are no publications specifically addressing tumor response after fractionated radiotherapy in SEGA. We found reports of a total of 34 patients with SEGA having received some form of irradiation (Table 1). Seven cases received fractionated adjuvant radiotherapy after complete resection, so in the absence of gross residual tumor after complete resection, no direct response to radiotherapy could be assessed in those cases [12]. The dose of fractionated radiotherapy was mentioned only in two publications: 25 × 2 Gy and 28 × 1.8 Gy; in neither study did SEGA further grow during the mentioned follow-up periods; 8 years and 1 year of follow-up, respectively [13, 14]. A total of 14 patients received single fraction RT, treated by the Gamma Knife. Taken together, it is clear that SEGA sometimes does respond to irradiation. However, the broad range of doses and fractionations administered, and the lack of detail in the reports, besides the short follow-up terms, are all elements that do not permit us to draw solid conclusions about the radiosensitivity of SEGA.

**Table 1:** Reported results of radiotherapy and radiosurgery for SEGA

Author (year)	Resection	FU (m)	Irradiation type	Treated	Dose (Gy)	Criteria
Kapp (1967) [49]	P	156	X-ray	1/1	N.S.	Clinically well
Sinson (1994) [50]	P	24	N.S.	0/1	N.S.	N.S.
Park (1997) [51]		12	GK SRS	2/2	6–25	Volume −70%/−80%
Matsamura (1998) [13]	P	96	RT	1/1	50	No growth
Sharma (2004) [52]	N.S.	N.S.	RT	7/N.S.	N.S.	1 recurred 22 years later
Wang (2006) [53]		67	GK SRS	0/3	15	No growth
Henderson (2009) [54]		48.2	GK SRS	1/3	12–20	N.S.
Park (2011) [55]	P	73	GK SRS	4*/6	11–20	N.S.
Jiang (2011) [12]	C		RT	7/7	N.S.	No recurrence
	P	16	RT	1/2	N.S.	N.S.
Gagliardi (2017) [56]		N.S.	GK SRS	1/N.S.	N.S.	N.S.
Azam (2017) [14]		12	RT	1/1	50.4	No growth
Present study		150	RT	2/2	60	Volume −80%

Prior resection: *P* partial, *C* complete, *GK SRS* Gamma Knife radiosurgery, *RT* fractionated radiotherapy

^*^One patient with formation of enlarging cyst was scored as non-responder and was salvaged by repeat SRS

### Malignant and potentially radiation-induced intracranial tumors in TSC

One single published case of a radiation-induced glioma was found, concerning a patient that was treated in 1987 with whole-brain radiotherapy (20 × 2 Gy) plus boost of 5 × 2 Gy with opposing lateral 8 × 9 cm fields [13]. The glioblastoma developed 8 years after radiotherapy in the temporal lobe, likely in the 50 Gy region. The irradiated SEGA did not grow during these 8 years. This article was referenced 28 times (Web of Science, retrieved 1 September 2017) and none of the referencing manuscripts mentioned other cases of radiation induced malignant brain tumor. Shepherd *et al*. described a meningioma to be the cause of death of one patient after 19 years of cranial irradiation; the meningioma was thought to arise as a secondary late side effect to radiation [15].

Seven more malignant gliomas were reported in patients with TSC that never received therapeutic radiation [16–22].

An article specifically looking at the incidence of malignant tumors in patients with TSC examined 240 patients over 14 years and concluded that there was only an increased risk in renal cancer but not of intracranial tumors [23].

## Discussion

While complete resection has been acknowledged as the first line treatment of SEGA, complete and safe surgical removal can be problematic [24, 25]. This is perhaps best illustrated by epidemiological studies that provide an unbiased perspective on real-life efficiency, safety, and cost of treatment. A study looking at three large US national healthcare claims databases examined the outcomes of SEGA surgery among patients with TSC who underwent SEGA surgery between 2000 and 2009 [26]. In 48.9% of the patients, postoperative complications were registered. The postoperative diagnosis of SEGA in 100% of the cases and high reoperation rates suggest that many patients had incomplete resection, regrowth, or contralateral regrowth. The authors concluded that alternative therapeutic strategies should be considered. Moreover, even when safe and complete removal is possible, the tissue damage that is inevitable in surgery, especially for bilateral deep-seated tumors, may lead to neurocognitive decline that could negate part of the positive neurocognitive effects of mass reduction and resolution or prevention of hydrocephalus.

Because of problems associated with surgery as first-line treatment for SEGA, the discovery of the activity of the immunosuppressive drug everolimus against SEGA (published in 2006, [27]) was met with considerable enthusiasm. Within 6 years, a multicenter randomized trial was published, demonstrating that at least 35% of the patients had a 50% or more reduction in SEGA volume after 2 years of treatment [6]. In a subsequent report of the same patient cohort that received at least one dose of everolimus either initially or after crossover, results and toxicity were reported up to almost 5 years of treatment [28, 29]. The median change in SEGA volume after 12 months was −37.8%, and this did not improve much, staying below −50% during continued treatment [29]. This somewhat disappointing observation is especially remarkable because 30% of the cases were crossovers from the placebo arm and thus may still have been in the initial response rather than the extended phase of response to everolimus. In this line, the majority of responses occurred within the first few months, the mean time to response was a short 5.32 months, and no further responders were counted beyond approximately 2.5 years. Of the 13 patients who progressed, 5 had first responded to treatment. Toxicity was significant, with 36% grade 3 adverse effects: stomatitis, pneumonia, and neutropenia, and 4.5% grade 4 adverse effects, which included neutropenia, pneumonia, febrile infection, gastroenteritis, and pneumothorax. Serious adverse events ascribed to treatment led to discontinuation in 9.9% of patients.

Significantly, SEGA usually grow back after cessation of everolimus [27, 30, 31], and control of epilepsy may dramatically depend on continuation of the drug [32], implying the necessity for continued, perhaps life-long, administration of the drug. Attention to long-term toxicity is justified because of metabolic (dyslipidemia, hypertriglyceridemia) [33, 34] and immunosuppressive side effects that can be life-threatening [35]. The cost of life-long treatment may be a problem as well, especially in developing countries [36]. These concerns have motivated some authors to perform resections to avoid having to continue the drug even during significant and ongoing responses to mTOR inhibitors [36]. Trelinska *et al*. studied dose-reduced maintenance therapy after at least 12 months of standard dosing, and concluded that SEGA volumes need to be closely monitored during reduced-dose maintenance everolimus therapy because the majority of SEGA increased in size, and that patients who did not have a significant response to standard doses should not be recommended for dose reduction [37].

Irradiation, while being a standard modality in the treatment of pilocytic astrocytoma, historically appears to have been excluded from the treatment options in SEGA. We found very little data to support the strong and widespread expert opinion against the use of fractionated stereotactic, or indeed any other form of irradiation in the treatment of SEGA. A single case of radiation-induced glioblastoma after whole brain radiotherapy has been systematically cited as an argument against the use of radiation, even when modern conformal radiation techniques are known to decrease the radiation burden to the healthy brain tissue by several orders of magnitude.

Because patients with TSC do not a priori have an increased risk of malignant intracranial tumors, there are no arguments to suggest that they would be more susceptible to radiation-induced intracranial tumors compared with other patient populations.

It could be argued that highly conformal radiotherapy techniques would not reduce the risk that the SEGA itself might become malignant. Indeed, in pilocytic astrocytomas, a few cases have been described that are consistent with radiation as a cause of malignant degeneration [37, 38]. Spontaneous malignant transformation has been described as well [39–44] and it is likely that the selection of tumors to receive radiotherapy may have induced an adverse bias. In grade 2 gliomas, it is well known that fractionated radiotherapy actually delays malignant transformation. Furthermore, clinically malignant behavior of SEGA itself appears to be exceedingly rare, and only three cases have been mentioned in publications, none of them after therapeutic radiation [45–47].

The bilateral SEGAs that we treated before everolimus was in use, with FSRT to a dose of 30 × 2 Gy, reacted slowly and progressively over a period of 8 years and decreased to 20% of their original volume. Such slow responses are common in low-grade or benign intracranial tumors and have previously led to incorrect conclusions regarding radioresistance of meningioma, for example.

Remarkably, after the start of everolimus for extracranial manifestations of TSC, rapid acceleration of the volumetric response of one of the SEGA occurred, suggesting that the irradiated SEGA may not have been fully inactivated by radiation and that the response of SEGA to everolimus likely involves different and perhaps complementary mechanisms of cell kill, potentially involving apoptosis, as has been described in in vitro experiments and xenografts [48].

While in our patient, radiation was delivered as the initial treatment for SEGA, followed by everolimus, opposite sequencing of these treatments could be more advantageous. First, the response to everolimus appears to be faster than to radiotherapy and could thus be more effective to avoid or perhaps even treat volume-dependent complications such as hydrocephalus. Second, a smaller radiation target would decrease radiation burden to the surrounding healthy brain.

It should be kept in mind that the goal of FSRT in this setting could be merely to prevent regrowth, and that further volume reduction may not even be required.

## Conclusion

Surgical resection is currently the treatment of choice for SEGA, and both US and European drug agencies approve treatment by everolimus only when resection is not possible.

There is, however, an unmet need for a non-surgical treatment option for a subset of SEGA, as illustrated by the considerable enthusiasm about the mTor inhibitor everolimus. Quite reliable and rapid responses are seen on initiating everolimus, and it has even been used in patients with hydrocephalus for symptom relief. To maintain tumor control, however, life-long maintenance treatment may perhaps be necessary, leading to concerns about continued patient exposure to its immunosuppressive action and metabolic side effects as well as the accumulated cost of therapy.

Irradiation appears to have been excluded from the therapeutic arsenal for SEGA without good reason and has never been really studied. The long-term favorable outcome of the bilateral SEGA that we treated with FSRT demonstrates that these tumors do respond slowly and progressively to fractionated radiotherapy, similar to other low-grade intracranial tumors. Moreover, the further response of one of the SEGA to intercurrent everolimus administration suggests an additive effect of radiation, and that the drug could be clinically exploitable. Indeed, responses to everolimus are particularly rapid, leading to reduced tumor volume, which facilitates more focused radiation that reduces the radiation-induced side effects as compared with pancranial irradiation. Patients who do not require continued systemic treatment for treating other disease manifestations of the tuberous sclerosis complex could potentially stop the drug after consolidation with FSRT.

Patients without hydrocephalus who require multiple or bilateral open surgeries may be a population in which induction treatment with everolimus followed by fractionated stereotactic radiotherapy could be an alternative to surgery.

## Data Availability

Data and materials are kept secure in an institution computer, and are available for sharing via the corresponding author.

## Abbreviations

SEGA: Subependymal giant cell astrocytoma
TSC: Tuberous sclerosis complex
FSRT: Fractionated stereotactic radiotherapy
